# ﻿Two new species of Synotaxidae from eastern China (Araneae, Synotaxidae)

**DOI:** 10.3897/zookeys.1248.150650

**Published:** 2025-08-04

**Authors:** Zhong-Jing Wang, Yan-Bin Yao, Xiao-Ting Lyu, Ke-Ke Liu, Yu-Cheng Lin

**Affiliations:** 1 Key Laboratory of Jiangxi Province for Biological Invasion and Biosecurity, College of Life Science, Jinggangshan University, Ji'an 343009, Jiangxi, China Jinggangshan University Ji’an China; 2 Jinshan College of Fujian Agriculture and Forestry University, Fuzhou 350007, Fujian, China Jinshan College of Fujian Agriculture and Forestry University Fuzhou China; 3 Key Laboratory of Bio-resources and College of Eco-environment (Ministry of Education), College of Life Sciences, Sichuan University, Chengdu 610064, Sichuan, China Sichuan University Chengdu China

**Keywords:** Family distribution, *Hamus*, identification key, mesh web spiders, taxonomy, *Tekellina*

## Abstract

This paper describes two new synotaxid members from eastern China: the species *Hamusqingming* Yao, Liu & Lin, **sp. nov.** (♂, ♀, Fujian) and *Tekellinazhangziqingi* Yao, Liu & Lin, **sp. nov.** (♂, ♀, Jiangxi). Diagnoses, detailed descriptions, and illustrations are provided for both species. The geographic distributions of five Synotaxidae genera are discussed.

## ﻿Introduction

Synotaxidae Simon, 1895 is a relatively small and lesser-known family of spiders that typically constructs webs varying in shape from an irregular sheet to an inverted bowl to a lattice-like structure (Jocqué and Dippenaar-Schoeman 2006). The composition of this family has changed considerably since its inception. Forster et al. (1990) reviewed and diagnosed Synotaxidae, delimiting ten genera within the family. Using a standard target-gene approach to explore the phylogenetic relationships among ecribellate orb-weaving spiders, Dimitrov et al. (2017) redefined Synotaxidae to include only *Synotaxus* Simon, 1895, while the remaining genera previously assigned by Forster et al. (1990) were transferred to Physoglenidae Petrunkevitch, 1928. Subsequently, one genus from Theridiidae Sundevall, 1833 (*Tekellina* Levi, 1957) and three genera from Nesticidae Simon, 1894 (*Gaucelmus* Keyserling, 1884, *Hamus* Ballarin & Li, 2015, and *Nescina* Ballarin & Li, 2015) were also transferred to Synotaxidae by Ramírez et al. (2022). The placement of the latter three genera in Synotaxidae was confirmed by Eskov and Marusik (2023).

The genus *Hamus* was originally classified within Nesticidae until its transfer to Synotaxidae by Ramírez et al. (2022), a taxonomic revision subsequently corroborated by Eskov and Marusik (2023). The genus *Tekellina* had uncertain familial placement following Marusik and Omelko (2017), until its formal transfer from Theridiidae to Synotaxidae by Ramírez et al. (2022).

Globally, Synotaxidae currently comprises five genera and 38 described species (WSC 2025), among which China and adjacent regions exhibit particularly high biodiversity, accounting for three genera and 12 documented species: China harbors seven endemic species, one transboundary species distributed across China and Laos, another transboundary species spanning China and Japan, plus one species each in Singapore, Philippines, and the Russian Far East.

During a recent examination of spider collections from eastern China, we found two synotaxid species belonging to *Hamus* and *Tekellina* and propose them as new species. The aim of this paper is to provide detailed diagnoses and descriptions for both new spider taxa and to survey the distribution of the family and genera.

## ﻿Materials and methods

Specimens were examined using a Jiangnan SZ6100 stereomicroscope with a KUY NICE CCD camera. Male and female copulatory organs were dissected and examined in 80–85% ethanol. The endogynes were cleaned with pancreatin (Álvarez-Padilla and Hormiga 2007). All specimens were photographed with an Olympus CX43 compound microscope with a KUY NICE CCD camera. For SEM photographs, the specimens were dried under natural conditions, sprayed with gold with a small ion-sputtering apparatus (ETD-2000), or were uncoated, and then photographed with a Zeiss EVO LS15 scanning electron microscope.

All measurements were made using a stereomicroscope (AxioVision SE64 rel. 4.8.3) and are given in millimeters. Leg measurements are given as total length (femur, patella, tibia, metatarsus, and tarsus). All examined specimen materials are deposited in the Animal Specimen Museum, College of Life Science, Jinggangshan University, Ji’an, China (**ASM-JGSU**).

Terminology of the male palp follows Estol et al. (2024) and Lin et al. (2016). Abbreviations used in the text or figures are as follows:

### ﻿Male palp

**CC** terminal claw-like arm of conductor

**Con** conductor

**CM** mesal arm of conductor

**CP (I–II)** ventral processes of the conductor (I–II)

**DA** dorsal apophysis of paracymbium

**DP** distal processes of paracymbium

**Em** embolus

**LF** lateral cymbial furrow

**Pa** paracymbium

**TA** terminal apophysis of tegulum

**TA (I–II)** terminal apophysis (I–II) of tegulum

**Te** tegulum

**VA** ventral apophysis of the paracymbium

### ﻿Epigyne

**CD** copulatory duct

**CO** copulatory opening

**EP** epigynal plate

**FD** fertilization duct

**Spe** spermatheca

### ﻿Ocular area

**ALE** anterior lateral eye

**AME** anterior median eye

**MOA** median ocular area

**PLE** posterior lateral eye

**PME** posterior median eye

## ﻿Taxonomy

### ﻿Family Synotaxidae

Taxon classificationAnimaliaAraneaeSynotaxidae

Simon, 1895

89ADB2E0-C3A2-5FB1-9420-01854E8525BF

#### Comments.

Until recently, the family was considered monotypic and restricted to the Neotropical Realm (Table 1). Current studies have revealed greater diversity with the description of genus *Hamus* Ballarin & Li, 2015 and *Tekellina* Levi, 1957 from China, and the recognition of the non-monophyletic status of the original taxa (Lin et al. 2016; Lin et al. 2024). The biogeographic range now extends to the Oriental and Palearctic realms (Table 1).

**Table 1.** List of Synotaxidae species examined in this study, with their geographic distribution region and assigned biogeographic realms. Data sourced from the World Spider Catalog (WSC 2025).

Genus	Species	Geographic distributions	Biogeographic realm
*Gaucelmus* Keyserling, 1884	*G.augustinus* Keyserling, 1884	North, Central America, Caribbean	Nearctic
	*G.calidus* Gertsch, 1971	Mexico, Guatemala	Nearctic
	*G.cavernicola* (Petrunkevitch, 1910)	Jamaica	Neotropical
	*G.pygmaeus* Gertsch, 1984	Panama	Neotropical
	*G.strinatii* Brignoli, 1979	Guatemala	Neotropical
	*G.tropicus* Gertsch, 1984	Panama	Neotropical
*Hamus* Ballarin & Li, 2015	*H.bowoensis* Ballarin & Li, 2015	China	Oriental
	*H.cornutus* Lin, Ballarin & Li, 2016	China, Laos	Oriental
	*H.kangdingensis* Lin, Ballarin & Li, 2016	China	Oriental
	*H.luzon* Lin, Ballarin & Li, 2016	Philippines	Oriental
	*H.mangunensis* Lin, Ballarin & Li, 2016	China	Oriental
	*H.qingming* **sp. nov.**	China	Oriental
*Nescina* Ballarin & Li, 2015	*N.kohi* Lin, Ballarin & Li, 2016	Singapore	Oriental
	*N.minuta* Ballarin & Li, 2015	China	Oriental
*Synotaxus* Simon, 1895	*S.bonaldoi* Santos & Rheims, 2005	Brazil	Neotropical
	*S.brescoviti* Santos & Rheims, 2005	Brazil	Neotropical
	*S.ecuadorensis* Exline, 1950	Costa Rica to Ecuador	Neotropical
	*S.itabaiana* Santos & Rheims, 2005	Brazil	Neotropical
	*S.jaraguari* Souza, Brescovit & Araujo, 2017	Brazil	Neotropical
	*S.leticia* Exline & Levi, 1965	Colombia	Neotropical
	*S.longicaudatus* (Keyserling, 1891)	Brazil	Neotropical
	*S.monoceros* (Caporiacco, 1947)	Trinidad, Guyana, Brazil	Neotropical
	*S.siolii* Santos & Rheims, 2005	Brazil	Neotropical
	*S.turbinatus* Simon, 1895	Panama to Ecuador	Neotropical
	*S.waiwai* Agnarsson, 2003	Guyana, Brazil, Paraguay	Neotropical
*Tekellina* Levi, 1957	*T.araucana* Marusik, Eskov & Ramírez, 2022	Chile, Argentina	Neotropical
	*T.archboldi* Levi, 1957	USA, Mexico	Nearctic
	*T.bella* Marques & Buckup, 1993	Bolivia, Brazil	Neotropical
	*T.crica* Marques & Buckup, 1993	Brazil	Neotropical
	*T.guaiba* Marques & Buckup, 1993	Brazil	Neotropical
	*T.haosiwen* Lin & Li, 2024	China	Oriental
	*T.helixicis* Gao & Li, 2014	China	Oriental
	*T.huihangi* Lin & Li, 2024	China	Oriental
	*T.minor* Marques & Buckup, 1993	Brazil	Neotropical
	*T.miuda* Rodrigues & Estol, 2024	Brazil	Neotropical
	*T.miudinha* Rodrigues & Estol, 2024	Brazil	Neotropical
	*T.picurrucha* Rodrigues & Estol, 2024	Brazil, Argentina	Neotropical
	*T.sadamotoi* Yoshida & Ogata, 2016	China, Japan	Oriental
	*T.yoshidai* Marusik & Omelko, 2017	Russia (Far East)	Palearctic
	*T.zhangziqingi* **sp. nov.**	China	Oriental

### ﻿Genus Hamus

Taxon classificationAnimaliaAraneaeSynotaxidae

Ballarin & Li, 2015

21355AF3-702A-56D5-A97E-664AFCD09EE7

#### Comments.

The genus *Hamus* was initially assigned to Nesticidae and later transferred to Synotaxidae (Ramírez et al. 2022). This genus includes only five species, distributed in southern China, Philippines and Laos (WSC 2025). Currently, *H.luzon* Lin, Ballarin & Li, 2016 occurs in the Philippines, and all other species are distributed in China (WSC 2025).

##### ﻿Keys to *Hamus* species

###### ﻿Males (male of *Hamusluzon* unknown)

**Table:** 

1	Tegulum without terminal apophysis I (Lin et al. 2016: 10, fig. 1A−D)	***H.cornutus***
–	Tegulum with terminal apophysis I (Lin et al. 2016: 14, fig. 3D−H)	**2**
2	Terminal apophysis I of tegulum with a blunt and short process (Lin et al. 2016: 14, fig. 3D−H)	***H.kangdingensis***
–	Terminal apophysis I of tegulum with a slightly sharp and long process (Lin et al. 2016: 17, fig. 5A−D)	**3**
3	Terminal apophysis II of tegulum with strongly curved apex (Fig. 2A−F)	***H.qingming* sp. nov.**
–	Terminal apophysis II of tegulum without apex (Ballarin and Li 2015: 180, fig. 1A−D)	**4**
4	Wide terminal apophysis process I of tegulum with a blunt tip (Lin et al. 2016: 17, fig. 5A−D)	***H.mangunensis***
–	Narrow terminal apophysis process I of tegulum with a sharp point (Ballarin and Li 2015: 180, fig. 1A−D)	***H.bowoensis***

###### ﻿Females (females of *H.kangdingensis* and *H.mangunensis* unknown)

**Table:** 

1	Spermathecae pear-shaped or slightly pear-shaped (Lin et al. 2016: 11, fig. 2E−G)	**2**
–	Spermathecae round (Lin et al. 2016: 15, fig. 4D−F)	**3**
2	Copulatory ducts strongly twisted (Ballarin and Li 2015: 183, fig. 2A−C with copulatory ducts reported as ‘fertilization ducts’)	***H.bowoensis***
–	Copulatory ducts relatively straight (Lin et al. 2016: 15, fig. 4D−F)	***H.cornutus***
3	Spermathecae touching, and the epigynal plate width is 2.8 times smaller than the spermathecal diameter (Fig. 3A, B)	***H.qingming* sp. nov.**
–	Spermathecae slightly separated, and the epigynal plate width is 2.3 times smaller than the spermathecal diameter (Lin et al. 2016: 15, fig. 4D−F)	***H.luzon***

### ﻿ Hamus qingming

Taxon classificationAnimaliaAraneaeSynotaxidae

Yao, Liu & Lin sp. nov.

A4590332-79CD-5537-A2FF-EC4326AAF8EE

https://zoobank.org/A43B041F-7AAD-4B6F-9E37-A7C032F4ADE8

**Figure 1.**
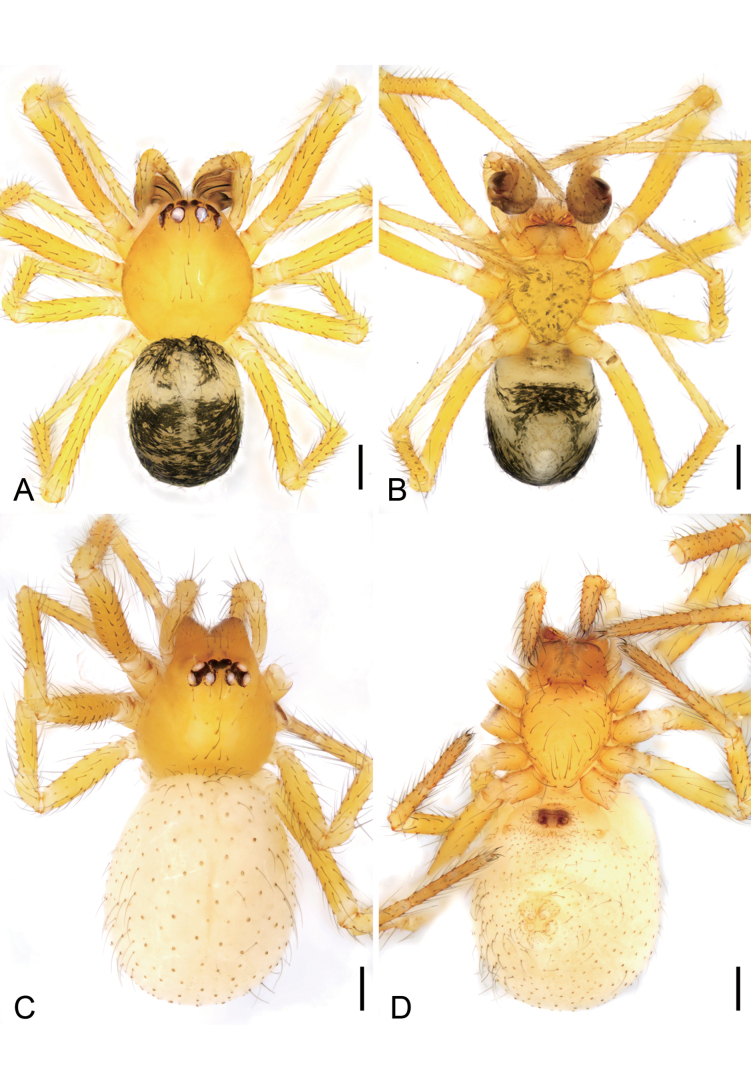
*Hamusqingming* sp. nov., male holotype and female paratype. A. Male habitus, dorsal view; B. Same, ventral view; C. Female habitus, dorsal view; D. Same, ventral view. Scale bars: 0.2 mm.

**Figure 2.**
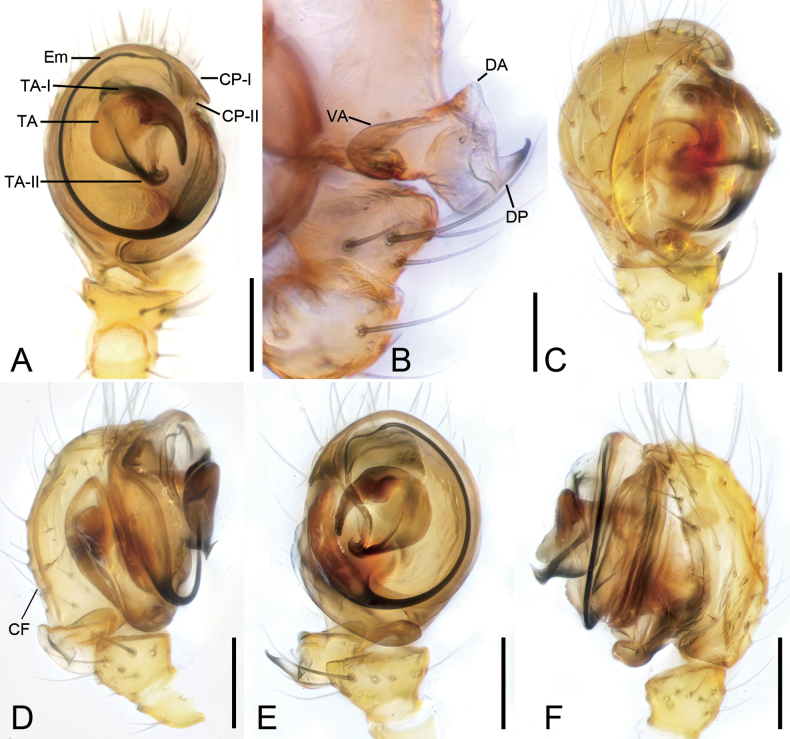
*Hamusqingming* sp. nov., male holotype. A. Left palp, ventral view; B. Paracymbuim of left palp, ventral view; C. Right palp, dorsal view; D. Same, retrolateral view; E. Same, ventral view; F. Same, prolateral view. Abbreviations: CP (I–II) – ventral processes of the conductor (I–II), DA – dorsal apophysis of the paracymbium, DP – distal processes of the paracymbium, Em – embolus, LF – lateral cymbial furrow, TA – terminal apophysis of tegulum, TA (I–II) – terminal apophysis (I–II) of tegulum, VA – ventral apophysis of the paracymbium. Scale bars: 0.1 mm.

**Figure 3.**
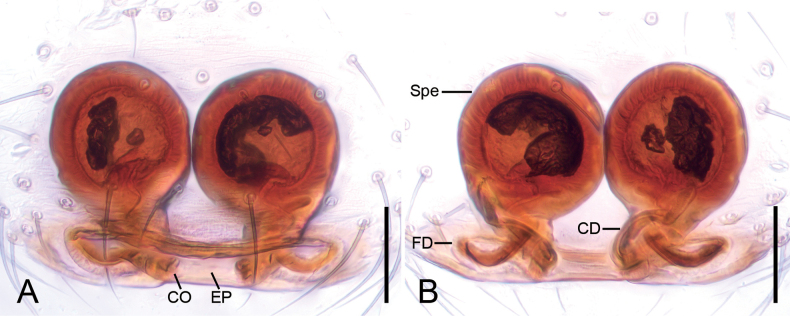
*Hamusqingming* sp. nov., female paratype. A. Epigyne, ventral view; B. Vulva, dorsal view. Abbreviations: CD – copulatory duct, CO – copulatory opening, EP – epigynal plate, FD – fertilization duct, Spe – spermatheca. Scale bars: 0.10 mm.

Figs 1 , 2 , 3 Common Chinese name: 清明钩蛛

#### Type material.

***Holotype*** ♂ and ***paratype*** 1 ♀. (Syn-01, ASM-JGSU). **CHINA**: • Fujian Prov., Zhangzhou City, Nanjing Co., Huboliao Nature Reserve, Huboliao area, 24°31'2.88"N, 117°14'53.47"E, 357 m a.s.l., 3.IV.2024, Y.B. Yao et al. leg.

#### Diagnosis.

The new species is similar to *H.bowoensis* Ballarin & Li, 2015 and *H.mangunensis* Lin, Ballarin & Li, 2016 (Ballarin and Li 2015: 180, fig. 1A−D; Lin et al. 2016: 17, fig. 5A−D) in having a clip-like terminal apophysis of the tegulum and a filamentous embolus, but can be separated from it by the large hook-shaped distal process of the paracymbium (vs. very tiny in *H.bowoensis* and *H.mangunensis*), a hook-shaped terminal apophysis II of the tegulum with a strongly curved apex (vs. without apex in both *H.bowoensis* and *H.mangunensis*), and the thick and finger-like ventral conductor processes I (vs. hook-like in *H.bowoensis* and *H.mangunensis*) (Fig. 2A−F). The female of the new species resembles that of *H.luzon* Lin, Ballarin & Li, 2016 (Lin et al. 2016: 15, fig. 4D−F) in having almost round spermathecae and the shorter, narrower fertilization ducts, but can be distinguished by the epigynal plate width 2.8 times smaller than the spermathecal diameter (vs. 2.3 times) and the touching spermathecae (vs. slightly separated) (Fig. 3A, B).

#### Description.

**Male** (holotype). Habitus as in Fig. 1A, B. Total length 1.28, carapace 0.66 long, 0.6 wide. Eye sizes and interdistances (Fig. 1A): AME 0.02, ALE 0.07, PME 0.06, PLE 0.07, AME−AME 0.06, AME−ALE 0.02, PME−PME 0.05, PME−PLE 0.05, AME−PME 0.03, AME−PLE 0.03, ALE−ALE 0.06, PLE−PLE 0.26, ALE−PLE 0.26. MOA 0.13 long, front width 0.11, back width 0.17. Endites trapezoidal, longer than wide, with dense setae arranged on the superomedial surface. Labium trapezoidal, wider than long, fused with sternum. Sternum (Fig. 1B) subtriangular shield-shaped, nearly as long as wide, lateral margin with scattered long setae. Legs (Fig. 1A, B): with abundant short setae; measurements: I 2.36 (0.72, 0.2, 0.21, 0.83, 0.4); II 2.42 (0.67, 0.29, 0.55, 0.45, 0.46); III 1.7 (0.38, 0.15, 0.49, 0.36, 0.32); IV 2.63 (0.82, 0.26, 0.66, 0.44, 0.45). Abdomen (Fig. 1A, B) 0.64 long, 0.53 wide.

***Coloration*** (Fig. 1A, B). Carapace yellow to light brown. Chelicerae yellow. Endites and labium yellowish to yellow. Sternum yellow to dark brown, with many dark brown spots on the surface. Legs yellow. Abdomen yellowish to dark, mottled, sub-anteriorly with a transversal light-yellow stripe.

***Palp*** (Fig. 2). Long lateral cymbial furrow on the retrolateral side of the cymbium. Paracymbium dorsal apophysis translucent and subtriangular, shorter than ventral apophysis, ventral apophysis tongue-shaped, as long as distal process of paracymbium, distal process hook-shaped, relatively large, slightly sclerotized. Terminal apophysis of tegulum well-developed and strongly sclerotized, with 2 curved processes forming together clip-like structure, terminal apophysis I of tegulum fang-like, terminal apophysis II of tegulum hook-shaped, with strongly curved apex. Conductor with 2 ventral processes distally, processes of the conductor I thick and finger-like, processes of the conductor II blunt and laminar, forming a C-shaped groove. Embolus filamentous, clockwise spiraling nearly 4/5 coil, arising from ~3 o’clock and ending at ~ 1 o’clock on tegulum.

**Female** (paratype). Habitus as in Fig. 1C, D. As in male, except as noted. Total length 1.79, carapace 0.63 long, 0.63 wide. Eye sizes and interdistances (Fig. 1C): AME 0.05, ALE 0.06, PME 0.05, PLE 0.07, AME−AME 0.03, AME−ALE 0.02, PME−PME 0.05, PME−PLE 0.03, AME−PME 0.04, AME−PLE 0.09, ALE−ALE 0.15, PLE−PLE 0.21, ALE−PLE 0.01. MOA 0.13 long, front width 0.11, back width 0.15. Legs (Fig. 1C, D): measurements: I 1.55 (0.75, 0.23, 0.57, missing); II 2.33 (0.6, 0.27, 0.56, 0.5, 0.4); III 1.96 (0.58, 0.21, 0.38, 0.21, 0.58); IV 2.81 (0.84, 0.29, 0.7, 0.45, 0.53). Abdomen (Fig. 1C, D) 1.16 long, 1.04 wide, with several long setae evenly.

***Coloration*** (Fig. 1C, D). Carapace yellow to reddish brown. Chelicerae, endites and labium reddish brown. Sternum yellow to reddish brown. Abdomen light yellow.

***Epigyne*** (Fig. 3). Translucent, more than 1.5 times wider than long. Spermathecae almost round, diameter subequal to half genital plate width, strongly sclerotized and touching each other. Copulatory ducts short, subequal to half spermathecal diameter, forming a C-shaped loop between the copulatory openings and the spermathecal base. Fertilization ducts narrow, connected to the base of the spermathecae and directed laterally.

#### Habitat.

In the tree near the ravine stream.

#### Distribution.

Known only from Fujian Province, China.

#### Etymology.

The name is taken from Qingming Festival (清明节), referring to the period of specimen collection during traditional Chinese Tomb-Sweeping Day; noun in apposition.

### ﻿Genus Tekellina

Taxon classificationAnimaliaAraneaeSynotaxidae

Levi, 1957

B77CA8F4-547B-5992-9236-6516DB0B3248

#### Comments.

The genus *Tekellina* was originally placed in Theridiidae, but its familial placement became uncertain following Marusik and Omelko (2017). It was subsequently transferred to Synotaxidae by Ramírez et al. (2022). This genus includes 14 species, mainly distributed in Brazil and East Asia (WSC 2025). Current records indicate four *Tekellina* species occur in China: *T.haosiwen* Lin & Li, 2024, *T.helixicis* Gao & Li, 2014, *T.huihangi* Lin & Li, 2024 and *T.sadamotoi* Yoshida & Ogata, 2016 (WSC 2025).

##### ﻿Keys to *Tekellina* species from China

###### ﻿Males

**Table:** 

1	Embolus with thick base (Yoshida and Ogata 2016: 15, figs 3–5)	***T.sadamotoi***
–	Embolus without thick base (Lin et al. 2024: 66, figs 57, 58)	**2**
2	Mesal arm of conductor without identical median ridge (Gao and Li 2014: 90, figs 72A, 74A, B)	***T.helixicis***
–	Mesal arm of conductor with identical median ridge (Lin et al. 2024: 66, figs 57, 58)	**3**
3	Mesal arm of conductor stubby and not reaching the terminal claw of the conductor (Fig. 7)	***T.zhangziqingi* sp. nov.**
–	Mesal arm of conductor long, reaching and surpassing the terminal claw of the conductor (Lin et al. 2024: 66, figs 57A, 58A)	**4**
4	Thick mesal arm of conductor with terminal lamellar process (Lin et al. 2024: 66, figs 57A, 58A)	***T.haosiwen***
–	Needle mesal arm of conductor with 3 terminal serrations (Lin et al. 2024: 67, figs 57B, 58B)	***T.huihangi***

###### ﻿Females

**Table:** 

1	Copulatory ducts thick (Yoshida and Ogata 2016: 15, figs 6, 7)	***T.sadamotoi***
–	Copulatory ducts thin (Gao and Li 2014: 90, figs 71C, D, 73A, B)	**2**
2	Distance of copulatory openings being wider than the distance between the retrolateral sides of spermathecae (Lin et al. 2024: 66, fig. 59A)	***T.haosiwen***
–	Distance of copulatory openings being shorter than the distance between the retrolateral sides of spermathecae (Lin et al. 2024: 70, fig. 59B)	**3**
3	Copulatory ducts with over 10 loops around spermathecae (Gao and Li 2014: 90, figs 71C, D, 73A, B)	***T.helixicis***
–	Copulatory ducts with 6 loops around spermathecae (Lin et al. 2024: 70, fig. 59B)	**4**
4	Spermathecal constriction nearly 5:5 (Fig. 5B)	***T.zhangziqingi* sp. nov.**
–	Spermathecal constriction nearly 3:7 (Lin et al. 2024: 70, fig. 59B)	***T.huihangi***

### ﻿ Tekellina zhangziqingi

Taxon classificationAnimaliaAraneaeSynotaxidae

Yao, Liu & Lin sp. nov.

4931A380-A44E-5AE8-9A8E-40A3EC39A6A0

https://zoobank.org/4A208104-CFDF-463E-852C-B2C62D84DC6F

**Figure 4.**
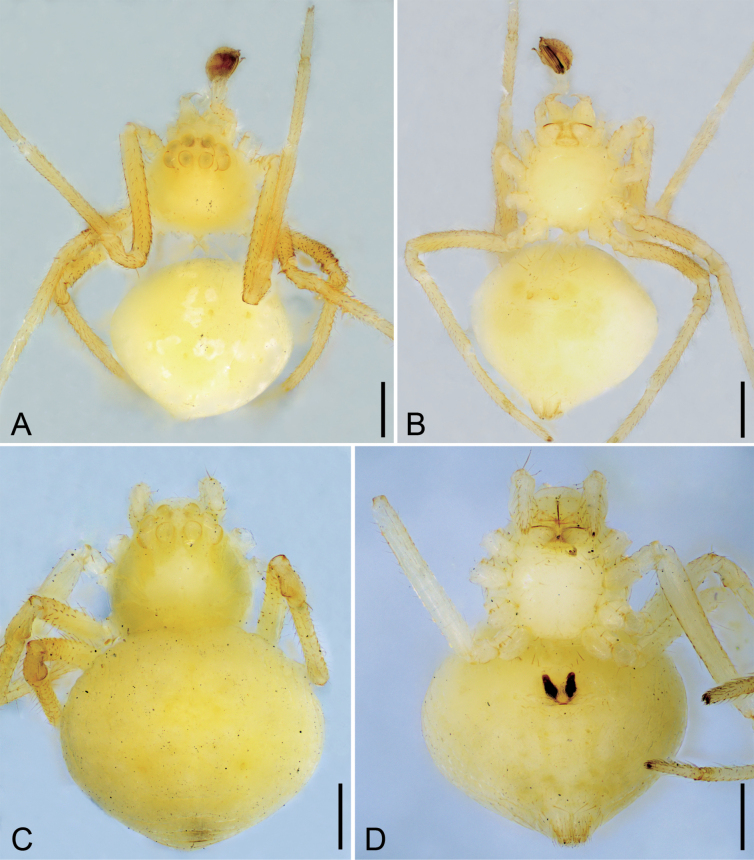
*Tekellinazhangziqingi* sp. nov., male holotype and female paratype. A. male habitus, dorsal view; B. Same, ventral view; C. Female habitus, dorsal view; D. Same, ventral view. Scale bars: 0.2 mm.

**Figure 5.**
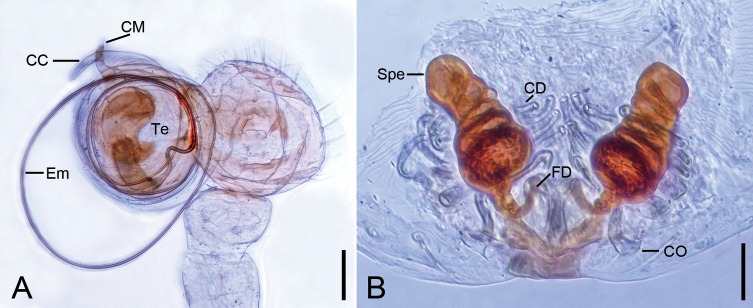
*Tekellinazhangziqingi* sp. nov., male holotype and female paratype. A. Right palp, ventral view; B. Vulva, dorsal view. Abbreviations: CC – terminal claw-like arm of conductor, CD – copulatory duct, CM – mesal arm of conductor, CO – copulatory opening, Em – embolus, FD – fertilization duct, Spe – spermatheca, Te – tegulum. Scale bars: 0.10 mm (A); 0.05 mm (B).

**Figure 6.**
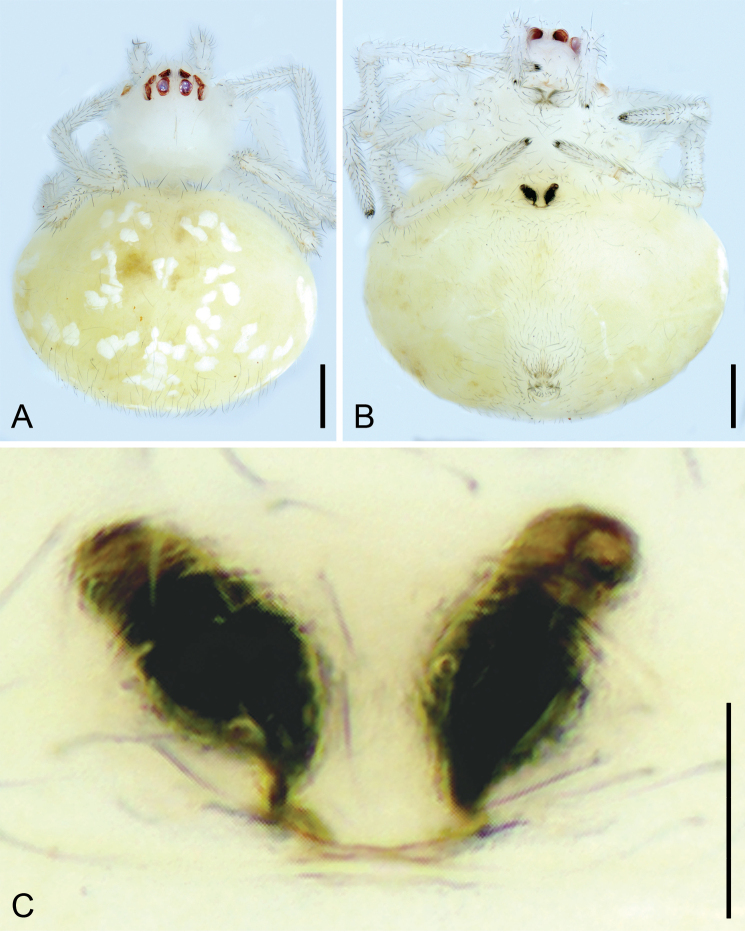
*Tekellinazhangziqingi* sp. nov., female paratype. A. Habitus, dorsal view; B. Same, ventral view; C. Detail of vulva, ventral view. Scale bars: 0.2 mm (A, B), 0.05 mm (C).

**Figure 7.**
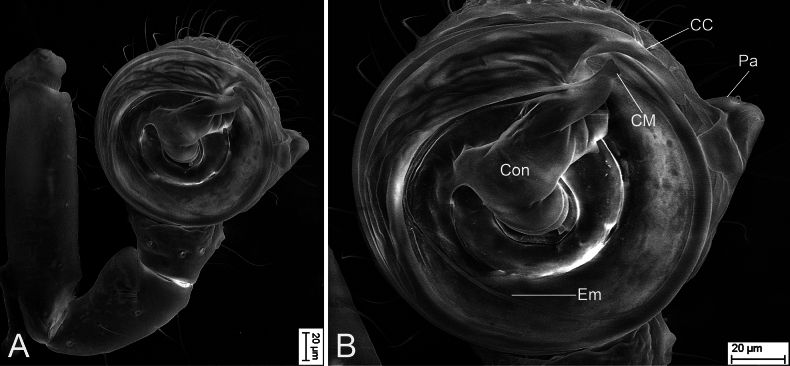
Scanning electron micrographs of *Tekellinazhangziqingi* sp. nov., male palp, holotype. A. Left palp, ventral view; B. Same, detail of bulb. Abbreviations: CC – terminal claw-like arm of conductor, Con – conductor, CM – mesal arm of conductor, Em – embolus, Pa – paracymbium.

**Figure 8.**
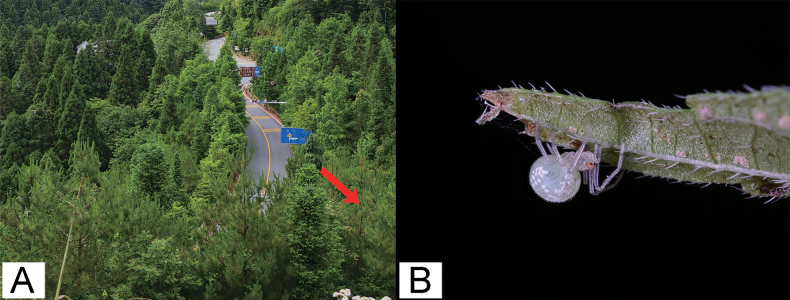
A. Habitat of *Tekellinazhangziqingi* sp. nov., red arrow shows the sampling point; B. Living specimen of *Tekellinazhangziqingi* sp. nov., female.

Figs 4 , 5 , 6 , 7 , 8B Common Chinese name: 张子清特克蛛

#### Type material.

***Holotype***: ♂, **CHINA**: • Jiangxi Prov., Ji’an City, Jinggangshan County Level City, Ciping Town, Jinggangshan Nature Reserve, Huangyangjie Scenic Spot, 26°38'9.6"N, 114°6'E, 1080 m a.s.l., 3.VIII.2014, K.K. Liu et al. leg. (Syn-02, ASM-JGSU); ***Paratypes***: • 1 ♀, same nature reserve, Zhufeng Scenic Spot, 26°31'58.8"N, 114°8'31.2"E, 708 m a.s.l., 1.VIII.2014, K.K. Liu et al. leg. (Syn-02, ASM-JGSU); 1 ♀, 17.VI.2025, Z.J. Wang and C.Z. Li leg. (ASM-JGSU-Ara-4501), other data same as holotype.

#### Diagnosis.

The male of the species resembles that of *T.helixicis* (Gao and Li 2014: 90, figs 72A, 74A, B) in having the enlarged and thumb-shaped paracymbium and the finger-like terminal claw-like arm of the conductor, but can be easily separated from it by the mesal arm of conductor with an identical median ridge and terminal lamellar process (vs. absent) (Figs 5A, 7A, B). The females can be easily distinguished from *T.helixicis* (Gao and Li 2014: 90, figs 71C, D, 73A, B) by the course of the loosely-spiral copulatory ducts surrounding the spermathecae (vs. tightly), the course of the copulatory ducts not reaching the apical part of the spermathecae (vs. reaching) and the spermathecae distally convergent (vs. nearly parallel) (Figs 5B, 6C).

#### Description.

**Male** (holotype). Habitus as in Fig. 4A, B. Total length 1.15, carapace 0.46 long, 0.42 wide. Eye sizes and interdistances (Fig. 4A): AME 0.06, ALE 0.05, PME 0.06, PLE 0.06, AME−AME 0.04, AME−ALE 0.01, PME−PME 0.03, PME−PLE 0.02, AME−PME 0.03, AME−PLE 0.06, ALE−ALE 0.1, PLE−PLE 0.03, ALE−PLE 0.01. MOA 0.13 long, front width 0.14, back width 0.14. Labium 3 times wider than long. Sternum (Fig. 4B) shield-shaped, slightly longer than wide. Legs (Fig. 4A, B): with neatly short setae; measurements: I missing; II 1.69 (0.49, 0.19, 0.45, 0.36, 0.2); III 1.29 (0.41, 0.16, 0.28, 0.22, 0.22); IV 1.57 (0.52, 0.14, 0.4, 0.29, 0.22). Abdomen (Fig. 4A, B) 2.52 long, 2.62 wide.

***Coloration*** (Fig. 4A, B). Carapace yellowish to brown. Chelicerae, endites, labium and sternum transparent to yellowish. Legs yellowish to yellow. Abdomen white to yellowish, with several white spots.

***Palp*** (Figs 5A, 7A, B). Paracymbium thumb-shaped. Conductor Y-shaped. Terminal claw of the conductor finger-like, approaching the hollow paracymbium. Mesal arm of the conductor stubby, narrow lamellar, with a truncated tip and not reaching the terminal claw of the conductor. Embolus filamentous and coiling, with four spirals.

**Female** (paratype). Habitus as in Fig. 4C, D, 6A, B. As in male, except as noted. Total length 1.18, carapace 0.39 long, 0.29 wide. Eye sizes and interdistances (Fig. 4C, 6A): AME 0.05, ALE 0.5, PME 0.06, PLE 0.06, AME−AME 0.05, AME−ALE 0.02, PME−PME 0.03, PME−PLE 0.02, AME−PME 0.05, AME−PLE 0.08, ALE−ALE 0.18, PLE−PLE 0.21, ALE−PLE 0.02. MOA 0.14 long, front width 0.13, back width 0.15. Sternum with sparse setae. Legs (Fig. 4C, D, 6A, B): measurements: I 1.57 (0.46, 0.18, 0.32, 0.29, 0.32); II 1.87 (0.62, 0.2, 0.56, 0.28, 0.21); III 1.43 (0.48, 0.13, 0.31, 0.29, 0.22); IV 1.77 (0.56, 0.16, 0.47, 0.32, 0.26). Abdomen (Fig. 4C, D, 6A, B) 0.79 long, 0.58 wide.

***Coloration*** (Figs 4C, D, 6A, B). Darker than males. Sometimes, the eyes with black annulations.

***Epigyne*** (Fig. 5B). Epigynal plate wider than long, weakly sclerotized and translucent. Copulatory openings separated by distance 2 × maximum spermathecal diameter. Copulatory ducts long, loose spirals surrounding spermathecae, not reaching the apical part of spermathecae. Fertilization ducts short, curved forward to mesal, directed medially. Spermathecae convergent, widely separate, constriction nearly 5:5.

#### Note.

Fig. 5A was imaged under a cover slip, thus altering the spatial relationship between the mesal arm of the conductor and the terminal claw of the conductor. However, the SEM images (in natural state) in Fig. 7 clearly demonstrate that the mesal arm of the conductor does not reach the terminal claw of the conductor. And in our newly collected paratype (Fig. 6), the female spermatheca also exhibits a significantly greater inclination angle than in *T.huihangi*, confirming this as a conspecific pairing rather than a variant.

#### Habitat.

In the low shrubs (Fig. 8A).

#### Distribution.

Known only from Jiangxi Province, China.

#### Etymology.

The species is named in honor of Zi-Qing Zhang, one of the famous figures of the extremely hard and bitter struggle in Jinggangshan; noun (name) in genitive case.

## ﻿Discussion

Some Synotaxidae spiders do not build webs. Their members predominantly occur in tropical regions but can also be found in some temperate zones. These spiders exhibit localized distributions, being strictly confined to warm and humid microhabitats with dense vegetation. Their current biogeographic pattern shows disjunct occurrences in tropical/subtropical zones of Asia, North and South America (absent in Antarctica, Oceania, Europe and Africa) (WSC 2025; Table 1). This pattern suggests strong ecological specialization, as demonstrated by:

1. *Gaucelmus* species occur from the Gulf of Mexico to the Caribbean. The South American species of *Gaucelmus* inhabit a wide range of climatic conditions in hot rainforest regions.
2. *Hamus* appears to be endemic to SE Asia, with members currently found only in tropical and subtropical localities in China, Laos, and the Philippines.
3. Only two *Nescina* species have been recorded from Asia, found in China and Singapore: *N.kohi* and *N.minuta*. However, due to the small sample size in this genus, the results should be regarded as preliminary.
4. All *Synotaxus* species are endemic to the Neotropical Realm, with species distributed in South America ranging between 20 °N and 22 °S, and surviving in habitats with temperatures ranging from 26 °C to 30 °C.
5. *Tekellina* exhibits a disjunct distribution across Southeast Asia’s temperate zones, southern North America, and broadly throughout South America. Most *Tekellina* species are arboreal with limited dispersal capacity, showing predominant endemism across their Southeast Asian and Neotropical Realm. However, unpublished records, such as those from Tasmania, Australia (Butt and Allison 1999), along with other potential occurrences on mainland Australia, suggest a wider distribution than currently documented. Their phylogenetically conserved microhabitat specificity and disjunct transcontinental distributions make these spiders valuable for biogeographic studies.
6. Current understanding of synotaxid biogeography remains limited; amber fossils from Baltic and German deposits (Oligocene) demonstrate significantly broader historical distributions in Synotaxidae than presently observed (Hormiga and Griswold 2014). While their minute size poses collection challenges, integrating paleontological and neontological data will better reveal their historical dispersal pathways.

## Supplementary Material

XML Treatment for Synotaxidae

XML Treatment for Hamus

XML Treatment for Hamus qingming

XML Treatment for Tekellina

XML Treatment for Tekellina zhangziqingi

## ﻿Additional information

### Use of AI

No use of AI was reported.

### Author ORCIDs

Zhong-Jing Wang https://orcid.org/0009-0000-6377-6840

Yan-Bin Yao https://orcid.org/0000-0002-2560-9299

Xiao-Ting Lyu https://orcid.org/0009-0000-5012-6101

Ke-Ke Liu https://orcid.org/0000-0001-7822-3667

Yu-Cheng Lin https://orcid.org/0000-0002-5054-0633

### Data availability

All of the data that support the findings of this study are available in the main text.
